# Kinetic data analysis for probiotic *Lacticaseibacillus rhamnosus* GG growth and *pH* drop in rice-based milk alternative

**DOI:** 10.3389/fmicb.2026.1791223

**Published:** 2026-04-24

**Authors:** Zuzana Matejčeková, Pavel Ačai, Tamara Brhelová Čmiková, Ĺubomír Valík

**Affiliations:** 1 Department of Nutrition and Food Quality Assessment, Institute of Food Science and Nutrition, Faculty of Chemical and Food Technology, Slovak University of Technology in Bratislava, Bratislava, Slovakia; 2 Department of Food Technology, Institute of Food Science and Nutrition, Faculty of Chemical and Food Technology, Slovak University of Technology in Bratislava, Bratislava, Slovakia

**Keywords:** growth description, *Lacticaseibacillus rhamnosus* GG, prediction, rice-based milk alternative, three-step kinetic data analysis approach

## Abstract

The objective of this study was to describe and predict the behavior of probiotic *Lacticaseibacillus rhamnosus* GG (LGG) in a rice-based milk alternative over a temperature range of 12 to 44 °C using the standard three-step kinetic data analysis process. According to the root-mean-square error (RMSE), the primary Baranyi and Roberts (BR) and the new logistic (NL) models described population growth with nearly identical fitting accuracy, with averages of 0.112 and 0.115, respectively. The secondary model for the specific growth rate (μ_max_), based on the outputs of the primary models (BR and NL), also produced very similar estimates of *T*_min_ values at 4.61 °C and 4.71 °C and *T*_max_ values at 49.67 °C and 49.69 °C, respectively. In general, the average coefficient of determination was *R*^2^ = 0.991 and *RMSE* = 0.185 for predicting LGG growth using the BR and NL models, each incorporating secondary models for the lag phase and μ_*max*_ (tertiary models). To model the *pH* kinetics, which exhibited a sigmoidal curve pattern, an approach analogous to that used for growth prediction was applied. The final *pH* prediction was performed using the Geeraerd model (GM_pH, T_), integrated with both secondary models and cardinal growth temperatures of *T*_min_ = 4.61 °C and *T*_max_ = 49.67 °C. This tertiary model demonstrated strong statistical performance, with *R*^2^ = 0.941 and *RMSE* = 0.386. The ability to predict the behavior of *L. rhamnosus* GG is valuable for designing and optimizing dairy-free matrices with probiotic potential.

## 1 Introduction

The increasing incidence of milk allergies and lactose sensitivity, and the growing popularity of a plant-based lifestyle, have significantly influenced food manufacturers to develop innovative dairy alternatives. These shifts not only address individual health and nutritional needs but also align with broader sustainability goals by reducing reliance on animal-derived products and lowering carbon emissions (Marchwińska et al., 2023).

Among the various plant-based milk substitutes, oat, and rice-based beverages have gained notable popularity due to their hypoallergenic properties, light taste, and ease of digestion (Marchwińska et al., 2023; Karoui and Bouaicha, 2024). Rice-based beverages offer several health benefits attributed to their micronutrient profile and bioactive compounds. They are relatively rich in selenium and magnesium, which are associated with improved immune function (Abou-Dobara et al., 2016). Furthermore, rice drinks contain phytosterols such as β-sitosterol and γ-oryzanol, known for their cholesterol-lowering, antihypertensive, antidiabetic, anti-inflammatory, and antioxidant effects (Faccin et al., 2009; Biswas et al., 2011). Typically produced from milled rice and water, rice-based milk alternatives are often fortified with calcium, vitamin D, and B12 to improve their nutritional value. Although they are generally high in calcium, their natural iron and zinc content is lower, potentially limiting their contribution to mineral intake without further fortification (Romulo, 2022). They also provide essential minerals such as magnesium, phosphorus, potassium, and sodium, which support electrolyte balance and metabolic functions. However, rice beverage is naturally low in protein (usually <1 g/100 ml) and lacks essential amino acids, with a Digestible Indispensable Amino Acid Score (DIAAS) of approximately 47, which is significantly lower than that of dairy or soy-based alternatives (Velangi and Savla, 2022; Karoui and Bouaicha, 2024). This requires fortification or mixing with other protein sources to meet nutritional standards comparable to cow's milk (Velangi and Savla, 2022).

From a compositional perspective, rice is rich in carbohydrates, primarily starch, which contributes to the viscosity and texture of rice-based beverages. To improve fluidity and reduce viscosity, enzymatic hydrolysis using α-amylase or glucoamylase is commonly employed to break down starch into simpler sugars (U.S. Department of Agriculture, 2021). This process not only enhances texture but also provides fermentable substrates for microbial growth. However, rice beverages are susceptible to rancidity due to endogenous lipases, particularly in rice bran, which hydrolyze lipids into free fatty acids, compromising shelf life and sensory quality (Bansal et al., 2023). Stabilization techniques such as heat treatment or enzyme inactivation are therefore essential (Liu et al., 2023).

*Lacticaseibacillus rhamnosus* GG (LGG), originally isolated from the human gut, is well-known for its probiotic efficacy and technological versatility (Yu et al., 2019). In dairy systems, LGG produces exopolysaccharides (EPS), which improve texture and mouthfeel. Recent studies have shown that LAB, including LGG, can also grow effectively in rice-based media after enzymatic hydrolysis, with EPS production occurring even at suboptimal temperatures (U.S. Department of Agriculture, 2021). Despite growing interest in applying lactic acid bacteria to plant substrates, the fundamental physiological response of strains such as *Lacticaseibacillus rhamnosus* GG in commercial rice beverages remains insufficiently characterized. In particular, baseline data describing growth kinetics and acidification patterns under controlled low-inoculum conditions are scarce, but such information is essential for establishing kinetic parameters for predictive microbiology. Further, plant-based matrices differ substantially from dairy substrates in nutrient composition, buffering capacity, and carbohydrate structure, which means that dairy-origin probiotic strains cannot be assumed to exhibit analogous kinetic behavior in these systems. By quantifying temperature-dependent growth and acidification across a biokinetic range of 12 to 44 °C and evaluating the suitability of primary and secondary predictive models, this study provides foundational insights that have not previously been available for rice beverages. These baseline data fill a critical knowledge gap and establish the groundwork for future research on probiotic behavior and process development in plant-derived systems.

## 2 Materials and methods

### 2.1 Bacterial strain and experimental setup

The bacterial strain used in this study was *Lacticaseibacillus rhamnosus GG* ATCC 53103. The strain is part of the collection of cultures at the Institute of Food Science and Nutrition of the Slovak University of Technology in Bratislava, Slovakia. It was stored at −30 °C in Man, Rogosa, and Sharpe (MRS) broth (Biokar Diagnostics, Beauvais, France) supplemented with 25% (v/v) glycerol. For experiments, LGG was subcultured in MRS broth at 37 °C under 5% CO_2_ for 24 h. The final inoculum was prepared by overnight incubation under the same conditions. The initial bacterial concentration for each experiment was adjusted to 2 log CFU.ml^−1^.

Experiments were carried out in parallel in 500 ml Erlenmeyer flasks containing 300 ml of pretempered commercially available, heat-treated rice beverage (DM Drogerie Markt GmbH + Co. KG, Karlsruhe, Germany), composed of water, 13% rice, sunflower oil, and sea salt.

The nutritional composition is detailed in Table 1. Each flask was inoculated with 3 ml of the prepared bacterial culture and thoroughly mixed. Static cultivations were carried out in duplicate at temperatures of 12, 15, 18, 21, 25, 30, 37, 40, and 44 °C under aerobic conditions. At predetermined intervals, 1 ml of the samples was taken and serially diluted in a solution of 0.85% NaCl (w/v) and 0.1% peptone (w/v) (Biolife, Milan, Italy) until the stationary phase was reached. Diluted samples were poured using MRS agar (Biokar Diagnostics) and incubated at 37 °C for 48 h under 5% CO_2_. Colony-forming units (CFU) were counted and expressed per ml of sample. *pH* was monitored throughout experiments using a WTW 720 pH meter (Inolab, Weilheim, Germany).

**Table 1:** Nutritional composition of rice beverage per 100 ml (DM Drogerie Markt GmbH + Co. KG, Karlsruhe, Germany).

Energy value	203 kJ/ 48 kcal
Fat	1.1 g
Of which saturated fatty acids	0.1 g
Carbohydrates	9.4 g
Of which sugars	6.7 g
Proteins	0.1 g
Salt	0.07 g
Fiber	0.1 g

### 2.2 Growth modeling

The traditional three-step kinetic data analysis approach (Whiting and Buchanan, 1993) was used to describe and predict the growth of *L. rhamnosus* GG over the biokinetic temperature range of 12–44 °C. Suitable primary and secondary predictive models were used in the first two steps of kinetic parameter estimation, employing non-linear regression analysis to minimize the errors between the observed and calculated data. In the first step, each growth curve is analyzed using the BR model (Baranyi and Roberts, 1994) with an average value of *q*_0_ (*h*_0_), a measure of the initial physiological state of cells, and the new logistic (NL; Fujikawa and Morozumi, 2006) model for the mean values of the curvature parameters *n* and *m*, related to the lag period and the stationary phase, respectively, to determine the relevant kinetic parameters (μ_*max*_and λ). Then, the extended Ratkowsky square root model (eSQRT_μ_; Ratkowsky et al., 1983) for the μ_*max*_ and the modified extended reverse square root relationship by Whiting and Buchanan (eSQRT_λ_; Whiting and Buchanan, 1993) for λ were applied to analyze the effect of temperature. Finally, the tertiary models constructed by integrating applied BR and NL models with the above-mentioned secondary models and using the parameters determined in the first two steps were used for forward predictions (Tarantola, 2005) of LGG growth in rice beverage at different temperatures.

#### 2.2.1 Primary predictive models

For the LGG growth description in a rice-based milk alternative, two predictive models, the BR and NL (Fujikawa and Morozumi, 2006), were applied in their differential forms with initial conditions.

*Baranyi and Roberts (BR) model:*


$$\begin{array}{c} \frac{dx}{dt}=\frac{1}{1+10^{-Q_{10}}}\frac{\mu_{\max}}{ln10}\left[1-10^{\left(x-x_{\max}\right)}\right] \end{array}$$ (1)

$$\begin{array}{c} \frac{dQ_{10}}{dt}=\frac{\mu_{\max}}{ln10} \end{array}$$ (2)

$$\begin{array}{c} t=0\;x=x_{0}\;Q_{10}=Q_{0,10} \end{array}$$

Where *x* = log *N* is the concentration of the microbial population in log CFU.ml^−1^; *t* is the incubation time; *Q*_10_ is the decimal logarithm of *q* the “theoretical critical substance,” which must be produced to enable growth; μ_*max*_ is the maximum specific growth rate, *x*_*max*_ = log *N*_*max*_ is the maximum concentration of the microbial population in log CFU.ml^−1^; *x*_0_ = log *N*_0_ is the initial concentration of the microbial population (log CFU.ml^−1^); Q_0, 10_ is the decimal logarithm of *q*_0_; the quantity of the initial “theoretical critical substance,” which is also a measure of the physiological state of the inoculum. During the lag phase, the population must perform some work to enter the exponential growth phase and multiply at the same rate μ_max_. This work is proportional to the quantity *h*_0_, indicating not only the physiological state of the cells, but also the suitability of the new environment for their growth and reproduction. Its connection with the initial “theoretical critical substance” *q*_0_ and the growth parameters μ_*max*_ and λ is expressed by the following equations:


$$\begin{array}{c} q_{0}=10^{Q_{0,10}} \end{array}$$ (3)

$$\begin{array}{c} h_{0}=ln\left(1+\frac{1}{q_{0}}\right) \end{array}$$ (4)

$$\begin{array}{c} \lambda =\frac{h_{0}}{\mu_{\max}} \end{array}$$ (5)

The value *h*_0_, a transformation of *q*_0_ and λ, is dependent on λ and μ_*max*_. The optimized parameters of the Baranyi model in its modified form are μ_*max*_*, Q*_0, 10_*, x*_0_, or *x*_*max*_. The regression algorithm may not reliably estimate the individual values of *Q*_0, 10_(q_0_) for some growth curves; in this case, the following recommended approach, based on the Baranyi hypothesis (Baranyi and Roberts, 1994). As the subculture procedures for *L*. *rhamnosus* GG in rice beverage were standardized under constant environmental conditions (assuming the same initial physiological state), the values *q*_0_ and *h*_0_ should be constant. Therefore, the parameter *Q*_0, 10_ = log *q*_0_ was fitted first from the individual microbial growth curves, and then the average value *Q*_0, 10*av*_ = log *q*_0, *av*_ ($h_{0,av}=ln\left(1+\frac{1}{q_{0,av}}\right)$) was used again as a fixed parameter to the same set of growth data. This approach of reanalyzing the growth curves has the advantage that the number of parameters evaluated in the Baranyi model (Equations 1, 2) is reduced from four to three: μ_*max*_*, x*_0_, and *x*_*max*_, and the lag phases are subsequently calculated from the relationship:


$$\begin{array}{c} \lambda =\frac{h_{0,av}}{\mu_{\max}} \end{array}$$ (6)

and allows us to obtain, for increasing μ_*max*_, in the suboptimal interval of rising temperatures, a gradual shortening of the λ, which is consistent with the expected behavior of microbial populations in a given environment. Logically, a temperature higher than the optimal growth temperature will reduce the growth rate and prolong the lag phase.

Then, the newly obtained kinetic parameters from *L*. *rhamnosus* GG growth curves, the μ_*max*_ and λ, were described with the suitable secondary model compatible with this Baranyi assumption to demonstrate the link relationship between λ, μ_*max*_ and q_0_ = q_0av_ (h_0_ = h_0av_) (Garre et al., 2025).

*New logistic (NL) model:*

The NL model, introduced by Fujikawa and Morozumi (2006), can be described after small modifications (Fujikawa and Kano, 2009; Fujikawa, 2010) as the ordinary differential equation with the initial conditions as follows:


$$\begin{array}{l} \;\frac{dx}{dt}=[1-10^{n(x_{\min}-x0}]\frac{\mu_{\max}}{ln10}[1-10^{m)x-x_{\max})}]=\\ \;[1-10^{n(0.999999x_{0}-x)}]\frac{\mu_{\max}}{ln10}[1-10^{m(x-x_{\max})}[\; \end{array}$$ (7)

$$t=0\;x=\;x_{0}$$

Where *x*_*min*_ = log *N*_*min*_ is related to the initial concentration of the microbial population *x*_0_ = log *N*_0_ (log CFU.ml^−1^) that needs to be almost equal to and slightly smaller than *x*_0_ (*x*_min_ = *x*_0_ – 1·10^−6^); *x* = log *N* is the concentration of the microbial population in log CFU.ml^−1^; *t* is the time; is the maximum specific growth rate; *x*_max_ = log *N*_max_ is the maximum concentration of the microbial population in log CFU.ml^−1^; *n* is the curvature parameter related to the period of lag phase; *m* is the curvature parameter of the growth deceleration from exponential to stationary phase.

The length of the lag phase of microbial growth can be calculated from the formula:


$$\begin{array}{c} \lambda =\frac{\frac{\mu_{\max}}{ln10}t_{ef}-\left(x_{ef}-x_{0}\right)}{\frac{\mu_{\max}}{ln10}} \end{array}$$ (8)

Where *t*_ef_ is the time chosen in the exponential growth phase; *x*_ef_ is the microbial concentration calculated at that time.

The NL (NL; Equation 7) model was applied again to the same LGG observed growth data set with the mean values of the curvature parameters *n* and *m*. A similar effect of linking lag phase changes and specific growth rates was observed over a temperature range of 12 to 44 °C, as with the primary BR model using the average value *q*_av_ (*h*_0av_). The number of optimized parameters was reduced from five to three: μ_*max*_, *x*_0_, and *x*_max_. The Fujikawa NL model is available only in differential form; therefore, all primary models were fitted using their differential equations to ensure mathematical consistency and comparability.

#### 2.2.2 Secondary predictive models

To account for fitting temperature dependence, the extended Ratkowsky model in its squared form (eSQRT_μ_, Equation 9) and its modification by Whiting and Buchanan (eSQRT_λ_, Equation 10) were used to describe μ_max_ and the lag phase:


$$\begin{array}{c} \mu_{\max}=b_{\mu }^{2}{\langle \left(T-T_{\min}\right)\left\{1-exp\left[c_{\mu }\left(T-T_{\max}\right)\right]\right\}\rangle }^{2} \end{array}$$ (9)

The regression coefficients *b*_μ_ and *c*_μ_ represent the model parameters, *T* is the temperature, *T*_*min*_ is the theoretical minimum temperature, and *T*_*max*_ is the maximum temperature.

According to Garre et al. (2025), the secondary model for the lag phase (λ) compatible with a constant *Q*_0, 10_ = log *q*_0, *av*_ or *h*_0_ = *h*_0, *av*_ in Equations 1, 2 that describes the link among λ, μ_*max*_, and *q*_0_ was used for the lag phase:


$$\begin{array}{c} \lambda =\frac{h_{0,av}}{\mu_{\max}}=\frac{\ln\left(1+\frac{1}{q_{0,av}}\right)}{b_{\mu }^{2}{\langle \left(T-T_{\min}\right)\left\{1-exp\left[c_{\mu }\left(T-T_{\max}\right)\right]\right\}\rangle }^{2}} \end{array}$$ (10)

Equation 10 can be simplified as follows for the calculated lag phase.


$$\begin{array}{c} \lambda =\frac{1}{b_{\lambda }^{2}{\langle \left(T-T_{\min}\right)\left\{1-exp\left[c_{\lambda }\left(T-T_{\max}\right)\right]\right\}\rangle }^{2}} \end{array}$$ (11)

where the initial guess of parameter b_λ_ can be: $b_{\lambda }=\sqrt{\frac{b_{\mu }^{2}}{\ln\left(1+\frac{1}{q_{0,av}}\right)}}=\sqrt{\frac{b_{\mu }^{2}}{h_{0,av}}}=b_{\mu }\sqrt{\frac{1}{h_{0,av}}}$

and for *c*_λ_ = *c*_μ_.

#### 2.2.3 Growth prediction models

Two tertiary models that integrate both the primary BR and NL models (Equations 1, 2, 7, respectively) with the secondary models (Equations 9, 10), were used for the parallel prediction of LGG growth in rice beverage.

#### 2.2.4 Correction factor

To assess the impact of the nutritive potential of various substrates on microbial growth, a correction factor (*c*_f_) can be employed. It is defined as a quantitative ratio between a representative secondary model parameter that characterizes the growth of a specific microorganism across different substrates under identical conditions. In this context, broth or milk can be considered a reference substrate for optimal bacterial growth. As demonstrated by Buss da Silva et al. (2017), one such representative growth parameter is the coefficient *b* from the SQRT model applied to *Bacillus cereus* grown in reconstituted infant formula and standard culture broth. Similarly, μ_*opt*_ from the Cardinal Temperature (CT) model proposed by Rosso et al. (1993) was applied to calculate the correction factor for plant-based beverages (PBB):


$$\begin{array}{c} \mu_{\max}=\mu_{opt}\;.\;\frac{\left(T-T_{\max}\right)\;.\;{\left(T-T_{\min}\right)}^{2}}{\left(T_{opt}-T_{\min}\right)\;.\;\left[\left(T_{opt}-T_{\min}\right)\;.\;\left(T-T_{opt}\right)-\left(T_{opt}-T_{\max}\right)\;.\;\left(T_{opt}+T_{\min}-2\;.\;T\right)\right]} \end{array}$$ (12)

where *T*_*min*_ is the theoretical minimum temperature; *T*_*max*_ is the maximum temperature; *T*_*opt*_ is the optimal temperature for μ_*opt*_

Accordingly, the correction factor was expressed as:


$$\begin{array}{c} c_{f}=\frac{b^{\left(PBB\right)}}{b^{\left(Milk\right)}} \end{array}$$ (13)

or


$$\begin{array}{c} c_{f}=\frac{\mu_{opt}^{\left(PBB\right)}}{\mu_{opt}^{\left(Milk\right)}} \end{array}$$ (14)

Where *b*^(PBB)^ and *b*^(Milk)^ are coefficients of the SQRT model for suboptimal growth of *L. rhamnosus* GG in plant-based beverages and milk, respectively. $\mu_{\text{opt}}^{\left(\text{PBB}\right)}$ is the optimal specific growth rate of *L. rhamnosus* GG in rice or oat beverage, and $\mu_{\text{opt}}^{\left(\text{Milk}\right)}$ is the corresponding value in milk.

In this study, both types of correction factors were applied to compare the nutritive potential of plant-based beverages with that of milk. The μ_opt_ values of *L. rhamnosus* GG in oat beverages and milk were obtained from previously published sources, Valík et al. (2008), 2013) for UHT milk and Matejčeková et al. (2025) for oat beverage.

### 2.3 *pH* changes description and prediction during *L. rhamnosus* GG growth

For the description and prediction of the *pH* experimental data change in a rice beverage, which shows a sigmoidal curve pattern throughout LGG growth but with a noticeable delay in the lag phase at the same temperature, an analogous kinetic data analysis approach as for the growth of microbial LGG in a rice-based milk alternative is applied. In the first step, the relevant kinetic parameters, the maximum rate of *pH* drop in the exponential phase (*k*_*pH*_) for the given temperature, and the duration of the lag phase in *pH* (λ_pH_) are estimated using the Geeraerd-inspired *pH* model (GM_pH_) (Geeraerd et al., 2000) expressed as follows:


$$\begin{array}{c} \frac{dpH}{dt}=-\frac{1}{1+10^{-C_{10}}}\frac{k_{pH}}{ln10}\left[1-10^{\left(pH_{end}-pH_{0}\right)}\right] \end{array}$$ (15)

$$\begin{array}{c} \frac{dC_{10}}{dt}=-\frac{k_{pH}}{ln10} \end{array}$$ (16)

*t* = 0 *pH* = *pH*_0_*C*_10_ = *C*_0, 10_

Where *pH*_0_ is the initial *pH* value; *pH*_end_ is the final *pH* value in a stationary phase; *C*_10_ can be considered as the theoretical protective substance that must be inactivated to disrupt the buffering capacity of the rice beverage before the obvious *pH* drop occurs.

We then assumed that the shoulder length (λ_pH_) of the *pH* change during microbial growth is also related to the initial value of *C*_10_ (*C*_0, 10_) that represents not only the initial dimensionless quantity of the theoretical protective substance, but also the initial acidification state of the rice beverage.

Applying the mathematical parallelism between the description of microbial growth by the BR model (Equations 1–6) and *pH* description through the GM, the equations as follows may be written:


$$\begin{array}{c} c_{0}=10^{-C_{0,10}} \end{array}$$ (17)

$$\begin{array}{c} g_{0}=ln\left(1+\frac{1}{c_{0}}\right) \end{array}$$ (18)

$$\begin{array}{c} \lambda_{pH}=\frac{g_{0}}{k_{pH}} \end{array}$$ (19)

Where *c*_0_ is related to the initial value of *C*_0, 10_; *g*_0_ indicates the buffering capacity of the rice beverage and is just a different transformation of the initial value *c*_0_. The lag phase in *pH* depends on *c*_0_ (*g*_0_) and the maximum *pH* drop rate in the exponential phase (*k*_*pH*_).

The *pH* change in a rice-based milk alternative in a biokinetic temperature range of 12 °C to 44 °C was also fitted twice. For the second time, the average value *C*_0, 10*av*_ = log *c*_0*av*_ ($g_{0,av}=ln\left(1+\frac{1}{c_{0,av}}\right)$) was used as a fixed parameter for the same set of *pH* change data. The number of parameters evaluated in the primary Geeraerd-inspired *pH* model (Equations 15, 16) is reduced from four to three: *k*_pH_*, pH*_0_, and *pH*_end_, and the phases of lag in *pH* (*pH*) are subsequently calculated from the relationship:


$$\begin{array}{c} \lambda_{pH}=\frac{g_{0,av}}{k_{pH}} \end{array}$$ (20)

This will again allow us to examine the interrelationship between the rate of *pH* decline and the length of the *pH* lag phase at a given temperature, which is consistent with the expected behavior of buffering capacity of a rice beverage and acidification state of the microbial population. Appropriate secondary models are mathematically compatible with the assumption to demonstrate the link relationship between λ_pH_, *k*_pH_, and *c*_0_ = *c*_0av_ (*g*_0_ = *g*_0av_).

The effect of temperature on *k*_pH_ under the entire biokinetic temperature range was evaluated by the modified Ratkowsky-inspired pH square root model as follows:


$$\begin{array}{c} k_{pH}={\langle b_{k}\left(T-T_{\min}\right)\left\{1-exp\left[c_{k}\left(T-T_{\max}\right)\right]\right\}\rangle }^{f_{k}} \end{array}$$ (21)

Where *b*_k_, *c*_k_, and *f*_k_ are the regression coefficients; *T*_*min*_ and *T*_*max*_ are the theoretical minimum and maximum growth temperature, respectively, determined for the analysis of LGG growth by the BR model (Equation 9).

The Whiting and Buchanan–inspired pH-modified reverse square-root relationship was used to model the pH lag time (λpH) as a function of temperature.


$$\begin{array}{c} \lambda_{pH}=\frac{1}{{\langle b_{\lambda pH}\left(T-T_{\min}\right)\left\{1-exp\left[c_{\lambda pH}\left(T-T_{\max}\right)\right]\right\}\rangle }^{f_{k}}} \end{array}$$ (22)

Where *b*_λ*pH*_*, c*_λ*pH*_*, and f*_λ_ are the regression coefficients.

The tertiary model, which integrates the primary GM (Equations 15, 16) and secondary models (Equations 21, 22), was used for the prediction of the change in *pH* in rice beverage for the estimated parameters *b*_*k*_*, c*_*k*_*, f*_*k*_, *b*_λ*pH*_*, c*_λ*pH*_*, f*_λ_.

### 2.4 Goodness-of-fit indices and statistical model comparison

Statistical ‘Goodness-of-fit' indices (Equations 23–26) are used to evaluate the performance of the primary, secondary, and tertiary models applied to fit and predict the individual or the entire set of observation points according to Equations 1–22, respectively:


$$\begin{array}{c} SSE=\overset{n}{\underset{i=1}{\sum }}{\left(x_{i}^{\exp}-x_{i}^{cal}\right)}^{2} \end{array}$$ (23)

$$R^{2}=1-\frac{SSE}{SST}=\frac{\sum_{i=1}^{n}{\left(x_{i}^{\exp}-x_{i}^{cal}\right)}^{2}}{\sum_{i=1}^{n}{\left(x_{i}^{\exp}-x_{i}^{\exp}\right)}^{2}}$$ (24)

$$\begin{array}{c} RMSE=\overset{n}{\underset{i=1}{\sum }}\frac{{\left(x_{i}^{\exp}-x_{i}^{cal}\right)}^{2}}{n-p} \end{array}$$ (25)

Where SSE is the sum of squared errors, is $x_{i}^{\exp}$and $x_{i}^{cal}$ correspond to the given response values observed (experimental) and calculated (temperature or pH), respectively; *n* is the entire number of data points for the number of curves (k) of data responses (temperature or pH); *R*^2^ is the determination coefficient of fitting ($R_{f}^{2}$) and prediction ($R_{p}^{2}$), is the mean of all the experimental values; SST is the global sum of total squares; RMSE is the root mean square error of fitting (*RMSE*_f_) or prediction (*RMSE*_p_), which is a measure of residual variability for the entire fitted/predicted data set; *p* is the number of determined parameters.

The corrected Akaike information criterion (*AIC*_*C*_) was used to estimate the quality of both applied tertiary models for predicting LGG (BR_T_ and NL_T_ models) in a rice beverage. It accounts for changes in goodness-of-fit and the number of estimated parameters. A model with a lower *AIC*_*C*_ value is considered the most likely to be correct (Motulsky and Christopoulos, 2003).


$$\begin{array}{c} AIC_{C}=nln\left(\frac{SSE}{n}\right)+2\left(p+1\right)+\frac{2\left(p+1\right)\left(p+2\right)}{n-p-2} \end{array}$$ (26)

The commercial process engineering software Athena Visual Workbench (Stewart & Associates Engineering Software, Madison, WI; www.athenavisual.com) was applied for parameter estimation of the primary and secondary models, as well as for *pH* values and predictions through the constructed tertiary models, Microsoft Excel (Microsoft, Redmond, WA, USA) for the *SSE, SST, R*^2^, *RMSE* and *AIC*_*C*_ values.

## 3 Results

### 3.1 Primary modeling of growth and *pH*-value

#### 3.1.1 Growth curve fitting and prediction

**Figure 1:**
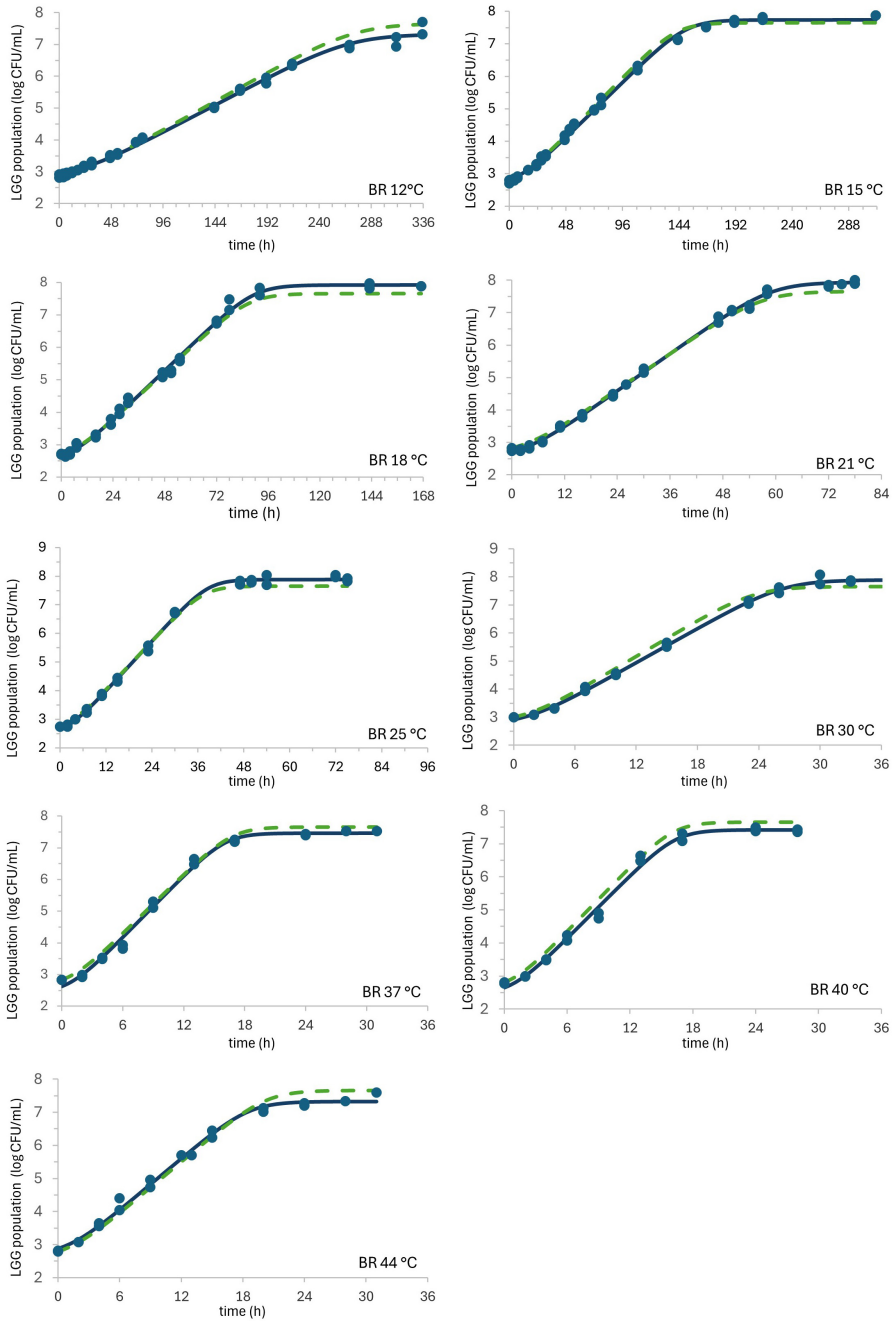
Baranyi and Roberts (BR) model fitted to data on *Lacticaseibacillus rhamnosus* GG growth at 12, 15, 18, 21, 25, 30, 37, 40, and 44 °C. Measured data points, fitting, and predicted values are represented by solid markers, solid lines, and dashed lines, respectively.

Both applied primary mathematical models (NL and BR) fitted/predicted the growth curves accurately across all data points (*n* = 246). The root mean square error of the fitting (*RMSE*_*f*_) was 0.118 log CFU.ml^−1^ and 0.122 log CFU.ml^−1^ using BR and NL models, respectively, with determination coefficient of fitting ($R_{f}^{2}$) of 0.997 (BR) and 0.996 (NL). For comparison, the calculated *RMSE*_*f*_ for the two models applied in the rice-based milk alternative is slightly less than that for the LGG growth description in the oat beverage (*RMSE*_*f*_ = 0.153), as the BR model with mean *q*_0_ = *q*_0*av*_ (*h*_0_ = *h*_0*av*_) was used (Matejčeková et al., 2025). The Huang model (HM; Huang, 2013) was also applied for the description and prediction (the results of the primary and secondary modeling are not shown) with even slightly better statistical indices for the fitting (*R*$R_{f}^{2}$= 0.997; *RMSE*_*f*_ = 0.103) than the BR and NL models used in this study, but apparently worse for the prediction of growth with the tertiary model ($R_{p}^{2}$ = 0.984; *RMSE*_*p*_ = 0.249 for the HM_T_ and $R_{p}^{2}$= 0.991; *RMSE*_*p*_ = 0.185 both for the BR_T_ and NL_T_). The residual errors of the observed and predicted data were also analyzed to evaluate the precision of the tertiary BR_T_ and NL_T_ models. More than 98% of the residual errors ranged between ±0.5 log CFU.ml^−1^ (BR_T_ = 99.2 %; NL_T_ = 98.4 %, while only 76.5 % were located within ±0.5 log CFU.ml^−1^ for the HM_T_ model). The *AIC*_*C*_ values for predicting growth and estimating the quality of each model were (−94.32, −94.28) for BR_T_ and NL_T_ models, respectively. As shown in Figure 1, LGG grew well across various isothermal temperatures, exhibiting a lag phase, an exponential phase, and a stationary phase. Given the substantial similarity between the growth patterns generated by the NL and those of the BR model, the former were excluded from individual graphical representations to avoid redundancy. In our rice beverage, the initial observed concentrations of *Lacticaseibacillus rhamnos* across the biokinetic temperature range were 2.81 ± 0.09 log CFU.ml^−1^ for the entire growth dataset. The estimated mean maximum cell concentration that reaches the stationary growth phase used then for prediction is 7.66 ± 0.27 log CFU.ml^−1^ and 7.67 ± 0.26 log CFU.ml^−1^ for BR and NL models, respectively. The estimated kinetic parameters describing LGG growth in our study are listed in Table 2 for the BR and NL models, respectively. The μ_max_ values of LGG in our rice beverage ranged from 0.041 h^−1^ (12 °C) to 0.604 h^−1^ (44 °C), with the highest values of 0.751 h^−1^ and 0.727 h^−1^ reached at 37 °C for the BR and NL models, respectively. At this temperature, the stationary phase was reached in the shortest time (around 24 h), accompanied by the minimal lag phases of 1.28 h and 0.90 h for the BR and NL models, respectively.

**Table 2:** Growth characteristics of *L*. *rhamnosus* GG in rice beverage using the Baranyi and Roberts (BR) model and the new logistic (NL) model across temperatures from 12 to 44 °C, including 95% confidence intervals.

BR					NL			
*T* (°C)	*μ_*max*_* (h^−1^)	*N_0_* (log CFU.ml^−1^)	*N_*max*_* (log CFU.ml^−1^)	λ _1_ (h)	*μ_*max*_* (h^−1^)	*N_0_* (log CFU.ml^−1^)	*N_*max*_* (log CFU.ml^−1^)	^1^λ (h)
12	0.041 ± 0.001	2.93 ± 0.06	7.33 ± 0.13	23.18	0.039 ± 0.001	2.98 ± 0.06	7.33 ± 0.14	17.29
15	0.082 ± 0.002	2.82 ± 0.05	7.74 ± 0.07	11.72	0.079 ± 0.003	2.81 ± 0.06	7.74 ± 0.09	7.43
18	0.146 ± 0.005	2.66 ± 0.08	7.92 ±0.12	6.57	0.140 ± 0.004	2.68 ± 0.10	7.92 ± 0.14	5.20
21	0.220 ± 0.005	2.71 ± 0.06	7.93 ± 0.07	4.37	0.212 ± 0.005	2.73 ± 0.06	7.93 ± 0.08	3.35
25	0.335 ± 0.013	2.66 ± 0.08	7.88 ± 0.07	2.86	0.320 ± 0.014	2.69 ± 0.09	7.88 ± 0.08	2.06
30	0.470 ± 0.015	2.91 ± 0.07	7.89 ± 0.06	2.04	0.455 ± 0.015	2.93 ± 0.08	7.89 ± 0.06	1.70
37	0.751 ± 0.051	2.63 ± 0.16	7.46 ± 0.16	1.28	0.727 ± 0.057	2.61 ± 0.18	7.46 ± 0.18	0.90
40	0.731 ± 0.050	2.65 ± 0.15	7.42 ± 0.16	1.31	0.707 ± 0.053	2.64 ± 0.17	7.42 ± 0.17	1.03
44	0.604 ± 0.036	2.88 ± 0.13	7.33 ± 0.14	1.59	0.581 ± 0.034	2.89 ± 0.12	7.33 ± 0.13	1.07

μ_max_, specific growth rate; N_0_, preliminary concentration of LGG; N_max_, concentration of LGG in the stationary phase; λ_1_, lag phase, values of λ_1_; , were calculated through the average value of q_0, av_ = −0.207; 0.960; values of ^1^λ;$\lambda =\frac{\left[\frac{\mu_{\max}}{\ln\;\left(10\right)}t_{ef}-\left(x_{ef}-x_{0}\right)\right]}{\frac{\mu_{\max}}{\ln\;\left(10\right)}}$, were calculated for the mean curvature parameters of the NLM: n = 12.663 and m = 0.958.

#### 3.1.2 Fitting and prediction of pH

**Figure 2:**
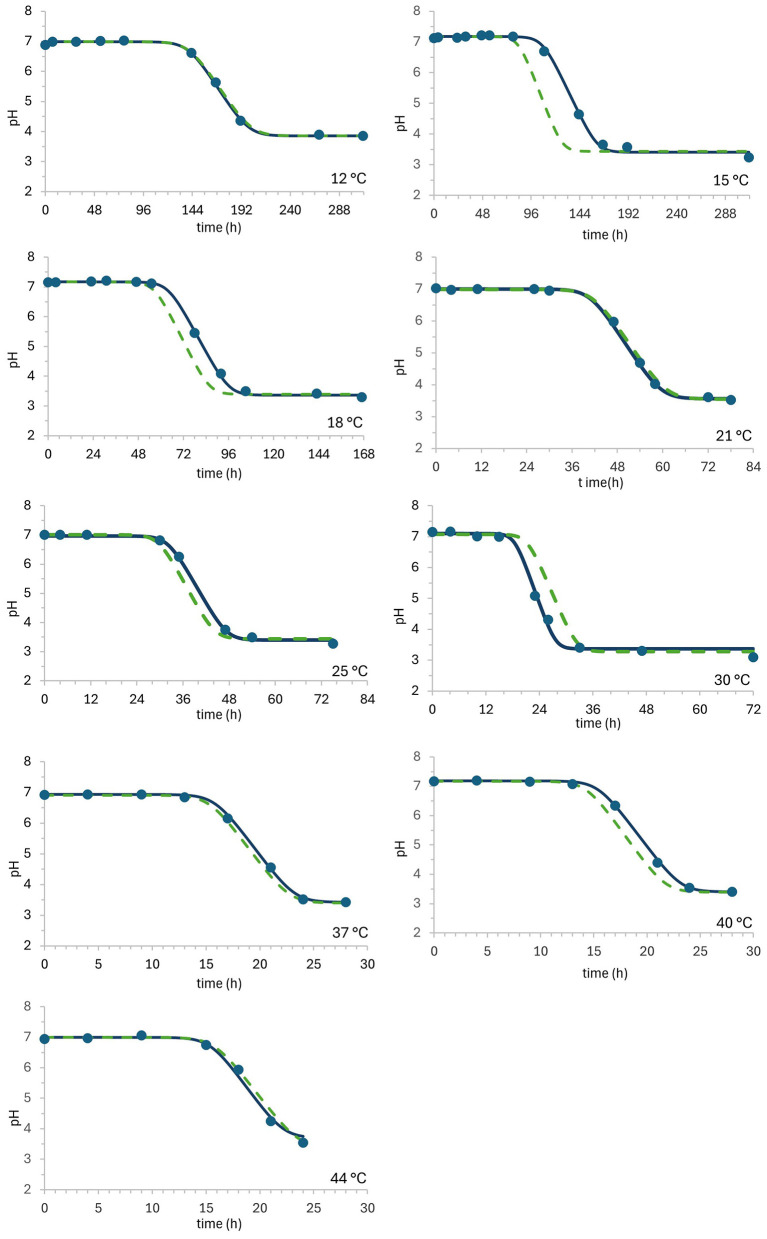
*pH* values during growth of *L. rhamnosus* GG in rice beverage at 12, 15, 18, 21, 25, 30, 37, 40, and 44 °C. Measured data points, fitted values, and predicted values are represented with solid markers, solid lines, and dashed lines, respectively.

Changes in *pH* appeared to follow a sigmoidal pattern over time, with three stages (lag, exponential, and stationary phase) (Figure 2), reflecting the buffering capacity of the rice beverage and the acidification capacity of LGG cells (Table 3). The initial *pH* of the rice-based milk alternative was 7.04 ± 0.11 for the given number of *pH* data responses. No appreciable *pH* changes were observed in the first stage (lag phase in *pH*), which is characterized by a noticeable delay in the lag phase within the same temperature range compared to growth curves. A temperature-dependent decline in the duration of the *pH* lag phase was determined, decreasing from 139.8 h at 12 °C to 15.2 h at 40 °C. The time required to reduce the *pH* from the initial observed value of 7.04 ± 0.11 to the *pH* value of approximately 4.0 varied significantly across different temperatures as LGG growth progressed into the exponential and stationary phases. The fastest *pH* decrease in rice beverage was calculated at temperatures of 40 °C (*k*_pH_ = 1.096 h^−1^) and 44 °C (*k*_pH_ = 1.096 h the μ_max_ value of LGG in rice beverage determined by the BR model was approximately 20% lower at 44 °C (μ_max_ = 0.604 h^−1^) than at temperature 37°C, at which μ_max_ was estimated (μ_max_ = 0.751 h^−1^) through the BR model, listed in Table 2. At the end of the third stage (stationary phase in *pH* change), the observed *pH* has decreased to 3.43 ± 0.25, whereas the LGG concentration remains approximately the same (stationary growth phase).

**Table 3:** Estimated parameters of the Geeraerd-inspired *pH* model describing the *pH* values in the rice beverage during the growth of *L. rhamnosus* GG in the temperature range of 12 to 44 °C, including 95% confidence intervals.

T (°C)	*pH*_0_	*pH*_end_	*k_*pH*_* (h^−1^)	^1^ $\lambda_{pH}(h)$
12	6.98 ± 0.06	3.86 ± 0.09	0.119 ± 0.002	139.8
15	7.17 ± 0.09	3.43 ± 0.17	0.158 ± 0.004	105.8
18	7.17 ± 0.04	3.39 ± 0.07	0.263 ± 0.003	63.5
21	6.99 ± 0.11	3.55 ± 0.15	0.408 ± 0.009	40.9
25	7.00 ± 0.12	3.44 ± 0.36	0.526 ± 0.050	31.7
30	7.08 ± 0.18	3.27 ± 0.37	0.907 ± 0.038	18.4
37	6.91 ± 0.03	3.39 ± 0.04	1.063 ± 0.017	15.7
40	7.17 ± 0.05	3.39 ± 0.09	1.096 ± 0.039	15.2
44	6.99 ± 0.16	3.38 ± 0.30	1.082 ± 0.144	15.4

pH_0_ , experimental initial pH value; pH_end_ , final pH value; k_pH_, rate constant for the decrease of pH; λ_pH_, lag phase duration in p, values of ^1^l_pH_; $\lambda_{pH}=\frac{g_{0,av}}{k_{pH}}=\frac{\ln\;\left(1+\frac{1}{c_{0,av}}\right)}{k_{pH}}$, are calculated through the average value of c_0, av_ = 7.244; $g_{0,av}=\ln\;\left(1+\frac{1}{c_{0,av}}\right)=$16.679.

### 3.2 Application of secondary temperature models

#### 3.2.1 Growth rate modeling

**Figure 3:**
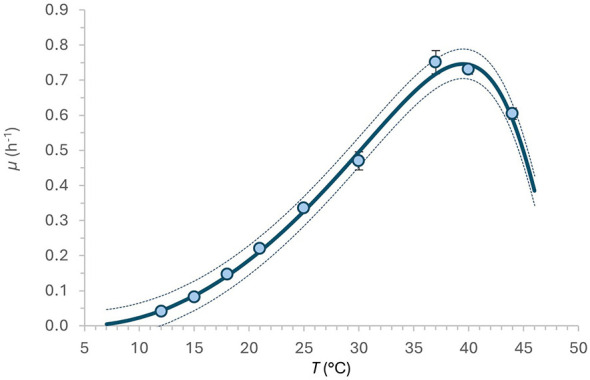
Experimental data fitting using the eSQRT model for the maximum specific growth rate (μ_*max*_). Dotted lines represent the range of ± 2*RMSE.

The secondary modeling results are shown in Table 4. The determined cardinal temperatures *T*_*min*_ are 4.61 °C and 4.71 °C, and *T*_*max*_ are 49.67 °C and 49.69 °C for the BR and NL models, respectively. The optimal temperature for LGG growth (*T*_*opt*_ = 39.11 °C) was determined using the gamma concept model (Zwietering et al., 1992 with μ_max_ values obtained by the BR model (Table 2). The estimated μ_*max*_ of LGG in a rice beverage varied between 0.043 at 12 °C and 0.601 h-1 at 44°C for the growth description of the BR model, with the highest value of 0.751 h^−1^ at 40 °C (*R*^2^ = 0.996). Similar μ_max_ obtained from the eSQRT_μ_ for the NL model growth description (0.042, 0.729, and 0.580 h^−1^ at observed temperatures 12, 40, and 44 °C, respectively) with *R*^2^ = 0.997 (Figure 3).

**Table 4:** Parameters of the modified square root model describing the temperature dependence of μ_*max*_ and λ, derived from two primary model fittings.

Model parameters	From BR		NL primary outputs	
*b_μ_* (h^−1^ °C^−1^)	0.028 ± 0.006	0.028 ± 0.001	0.0278 ± 0.005	0.033 ± 0.001
*T_*min*_* (°C)	4.61 ± 3.60	^*^4.61	4.71 ± 3.51	^*^4.71
*T_*max*_* (°C)	49.67 ± 2.92	^*^49.67	49.69 ± 2.80	^*^49.69
*c_*m*/λ_* (h^−1^ °C^−1^)	0.206 ± 0.138	0.222 ± 0.070	0.209 ± 0.136	0.225 ± 0.189
*R^2^*	0.996	0.999	0.997	0.994
***RMSE***	0.021	0.232	0.021	0.445

^*^Parameters T_min_ and T_max_ were fixed.

#### 3.2.2 Secondary modeling of pH decrease

**Figure 4:**
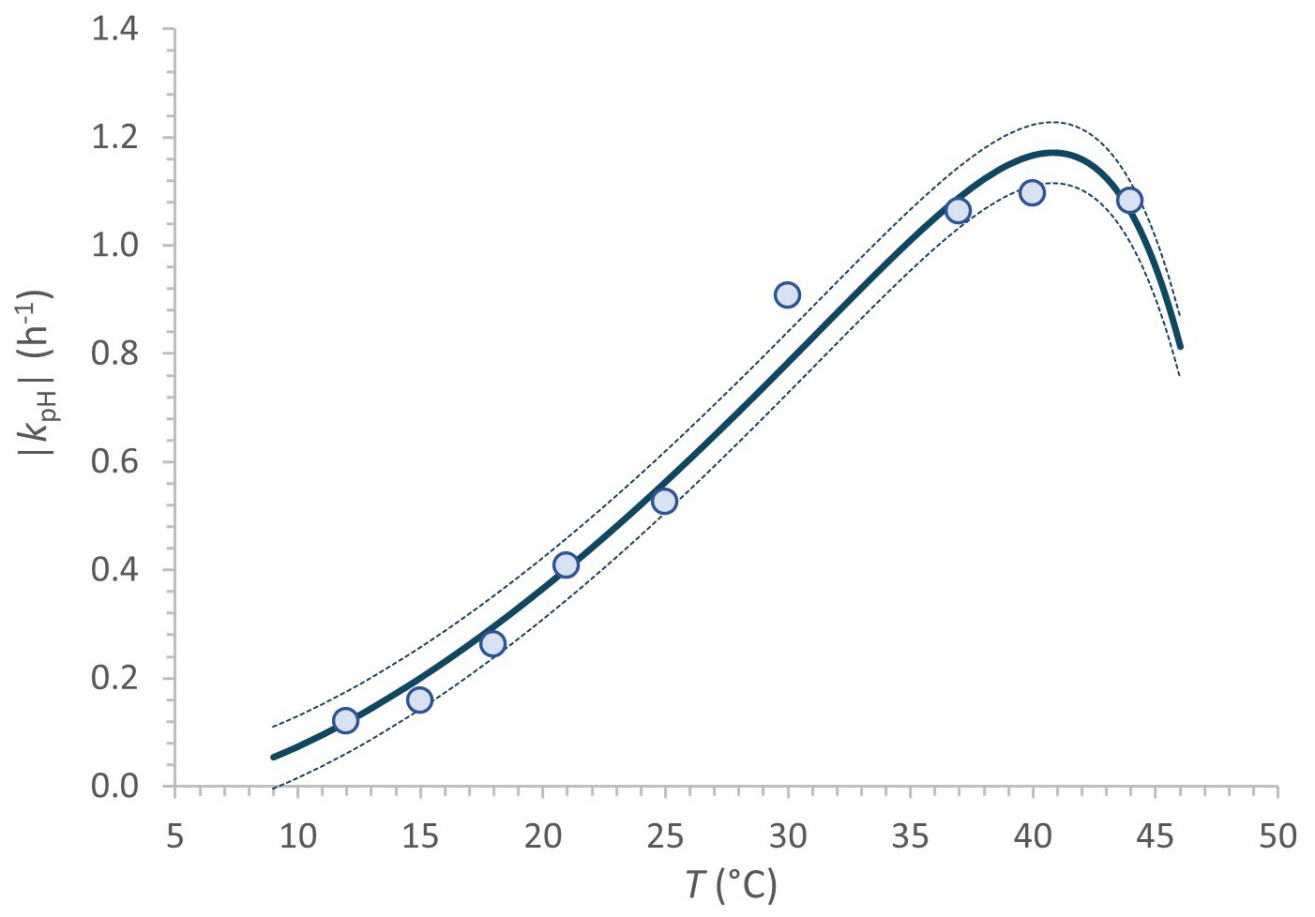
Application of the eSQRT model to the absolute rate constant of *pH* change during the growth of *L. rhamnosus* GG in rice beverage across the temperature range of 12 °C to 44 °C. Observed values are shown as solid markers, while the fitted curves represent model predictions.

Then, the suitable secondary models, the modified Ratkowsky-inspired square-root pH model (Equation 21) and the modified Whiting and Buchanan-inspired square-root pH model (Equation 22), were used to analyze the effect of temperature on *k*_*pH*_ and λ_*pH*_ (Table 5), respectively. Finally, the tertiary model for the *pH* prediction achieved an *RMSE* of 0.386 and an *R*^2^ value of 0.941 (Figure 4).

**Table 5:** Parameters of the modified Ratkowsky-inspired square root *pH* model (mRTK) and the modified Whiting and Buchanan-inspired square root *pH* model (mWB) with the 95% confidence intervals in conjunction with the Geeraerd-inspired *pH* model.

Model parameters	mRTK	mWB
*b_*k*_* (h^−1^ °C^−1^)	0.0338 ± 0.0031	-
*b _λ, *pH*_*(h^−1^ °C^−1^)	-	0.0042 ± 0.0029
*T_*min*_* (°C)	4.61	4.61
*T_*max*_* (°C)	49.67	-
*T_*max, pH*_*	-	49.67
*c_*k*_* (h^−1^ °C^−1^)	0.267 ± 0.098	-
*c_λ, *pH*_* (h^−1^ °C^−1^)	-	^*^0.438 ± 1.771
*f_*k*_*	1.538 ± 0.378	1.438 ± 0.300
*R^2^*	0.981	0.976

^*^The confidence interval of the parameter c _λ, *pH*_ is higher than its estimated value.

## 4 Discussion

The present study provides baseline insights into the temperature-dependent growth behavior and acidification dynamics of *Lacticaseibacillus rhamnosus* GG in a commercial rice-based beverage under low-inoculum conditions (*N*_0_ = 2.81 ± 0.09 log CFU.ml^−1^). However, industrial fermentations start with high inoculum levels, and controlled low initial densities are necessary to identify kinetic parameters with high sensitivity. Although predictive models such as the BR and NL are well established in dairy systems, their application to plant-based matrices remains limited. The low RMSE_f_ values and high R_f_^2^ obtained for both BR and NL models indicate that these primary models describe the LGG growth dynamics in the rice-based matrix with high precision. According to Mossel et al. (1995), acceptable deviations of the fitted RMSE for growth models typically range between 0.25 and 0.50, whereas values within 0.10 and 0.15 reflect particularly strong model performance. The RMSE_f_ values observed in this study fall well within this stricter range, further supporting the robustness of the modeling approach. The slightly lower RMSE_f_ values compared to those previously reported for LGG in an oat beverage (RMSE_f_ = 0.153), where the BR model with mean *q*_0_ = q_0av_ (h_0_ = h_0av_) was applied (Matejčeková et al., 2025), suggest that the rice beverage provided more consistent growth patterns under the tested conditions. The AIC_C_ values further support the suitability of the BR_T_ and NL_T_ models for predictive applications, indicating model reliability.

Almost the same initial LGG concentrations were experimentally obtained in the oat beverage (2.7 ± 0.11 log CFU.ml^−1^), while in the stationary phase, the optimized maximum population densities were 8.10 ± 0.38 log CFU.ml^−1^ (Matejčeková et al., 2025), showing that the oat beverage provided slightly better conditions for the growth of LGG than the rice beverage. LGG in the study by Kocková et al. (2013) increased from an initial level of 6.20 log CFU.ml^−1^ to 8.57 log CFU.ml^−1^ when fermenting rye flour (37 °C, 10 h). García-Escamilla et al. (2024) observed the viable LGG concentration of 9.72 ± 0.10 log CFU.ml^−1^ after 24 h at 37 °C in rye protein-enriched media, representing an increment of 2 logarithmic cycles. In black rice milk, the highest number of *L. acidophilus* (7.97 log CFU.ml^−1^) was reached after 24 h at 37 °C (Coşansu et al., 2021). The temperature-dependent growth behavior of LGG observed in this study is consistent with findings reported for other plant-based substrates. In the oat beverage, *L. rhamnosus* GG growth was characterized by μ_*max*_ values ranging from 0.038 h^−1^ to 0.602 h^−1^ at 12–44 °C, and minimal and maximal temperatures were estimated as follows: *T*_*min*_ = 4.2 °C and *T*_*max*_ = 47.8 °C (Matejčeková et al., 2025). A good performance of the Ratkowsky square root model on the growth of *L. rhamnosus* GG in milk was also shown by Valík et al. (2008). Matejčeková et al. (2019) reported a good prediction (*R*^2^ = 0.989) for *L. plantarum* across three substrates (UHT milk, lactose-free milk, and MRS broth). High population densities, maximum specific growth rates, and the comparison above illustrate that while plant-based matrices support LAB growth effectively, it is useful to consider the acidification behavior of the individual matrices. When comparing oat and rice-based milk alternatives, it follows that the *pH* lag phase in oat beverage was longer throughout the entire temperature range of LGG growth (12–44 °C), lasting 263.8 h at 12 °C to 20.3 h at 44 °C (Matejčeková et al., 2025) than in the rice beverage (Table 3). Coşansu et al. (2021) detected a *pH* of 4.86 after 18 h of incubation in white rice milk when the *L. acidophilus* population was over 6 log CFU.ml^−1^, with a final value of 4.27 (Δ*pH* = 3.36) after 40 h of incubation at 37 °C (*P* < 0.05).

### 4.1 Comparing lag phases for growth and *pH* decline

**Figure 5:**
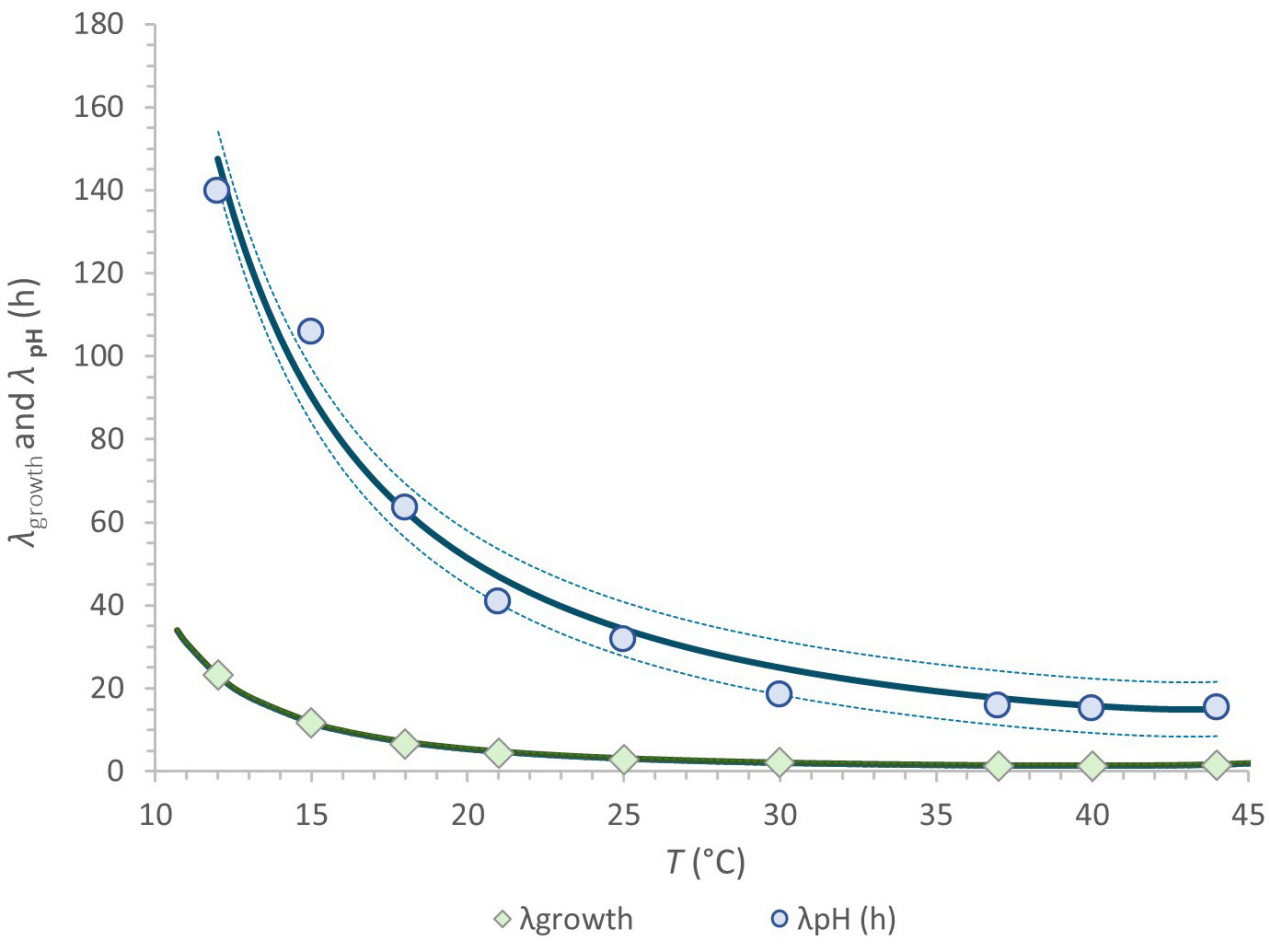
Comparison of *L. rhamnosus* GG adaptations in growth and metabolism using the eSQRT model applied to lag phase data for microbial growth and *pH* change in rice beverage across the temperature range of 12 °C to 44 °C. Observed values are depicted as solid markers; fitted curves represent model predictions, with dotted lines indicating standard errors of the fit.

Figure 5 presents a comparative analysis of the adaptation periods for growth and metabolism in rice-based beverages across a temperature gradient ranging from 12 to 44 °C. Supplementary Figure 1 further illustrates the time–temperature dependence, emphasizing the additional time required beyond the growth lag phase for metabolic processes to commence. This interval, inferred from *pH* dynamics, may reflect underlying regulatory mechanisms associated with metabolic shifts. Collectively, with the buffering capacity of the milk alternative, these observations suggest that the interplay between growth and metabolic lag phases could serve as a valuable criterion not only for strain characterization but also for optimizing fermentation strategies (Suissa et al., 2023).

### 4.2 Shift between *pH* and growth lag phases

It is widely acknowledged that measurements associated with the lag phase often exhibit greater variability than those related to growth rates (Valík et al., 2008). Nevertheless, in the present quantitative characterization of the *L. rhamnosus* strain, the observed growth and *pH* lag phase data appear sufficiently consistent to permit a comparative assessment of adjustment periods for both cellular proliferation and metabolic activation. *L. rhamnosus* GG, a well-documented probiotic strain frequently used as an adjunct culture in dairy fermentations, is known to undergo a metabolic transition following initial growth (Valík et al., 2013; Okur et al., 2025). Except for buffering capacity, the duration of transition between growth and *pH* lag periods may provide an additional phenotypic indicator of strain-specific adaptation under fermentation conditions. This parameter could therefore contribute to a more nuanced understanding of functional performance in complex food matrices. So, as metabolic byproducts accumulate, the *pH* can shift significantly after the lag phase.

From an industrial perspective, understanding these adaptation dynamics can help improve process control in non-dairy fermentations, where temperature fluctuations and substrate composition can significantly influence metabolic activity. Similar findings have been reported in studies on LAB in cereal-based substrates, which highlight the importance of metabolic lag as a determinant of acidification kinetics and sensory attributes (Charalampopoulos et al., 2002; Ziarno and Cichońska, 2021).

### 4.3 Growth correction factor

To evaluate the nutritive potential as an intrinsic characteristic of alternative plant milk beverages, this study used data on *L. rhamnosus* GG from previously published sources: Valík et al. (2008), 2013) for milk and Matejčeková et al. (2025) for oat beverage. Correction factors were calculated based on the regression parameter *b* from the suboptimal SQRT model (Buss da Silva et al., 2017) and the μ_opt_ value from the cardinal temperature (CT) model for *L. rhamnosus* GG in rice beverage compared to milk. The resulting correction factors were nearly identical: 0.373 and 0.371, respectively. This indicates that *L. rhamnosus* GG grows in rice-based beverage at approximately 40% of the rate observed in milk, suggesting a significantly lower nutritive potential of rice drink compared to milk. Importantly, both modeling approaches yielded consistent results.

For the oat beverage (Matejčeková et al., 2025), the correction factors were 0.227 (SQRT model) and 0.404 (CT model), showing a greater discrepancy between the two methods. Nevertheless, the μ_opt_ value suggests that the nutritive potential of the oat drink for *L. rhamnosus* GG is comparable to that of the rice drink, again, roughly 40% of milk's nutritive potential.

For other bacterial species, Buss da Silva et al. (2017) reported a correction factor of 0.67 for *Bacillus cereus* in reconstituted infant formula compared to BHI broth, based on the *b* parameter of the SQRT model. More recent data from Rockaya et al. (2026) using the CT model showed higher correction factors for *B. licheniformis* in almond (0.49), white almond (0.93), and coconut milk alternatives (0.87), all compared to BHI broth. These findings suggest that the nutritional potential of plant-based milk alternatives may be higher for spoilage spore-forming bacteria than for *L. rhamnosus* GG.

## 5 Conclusion

The comprehensive kinetic modeling approach applied in this study successfully described and predicted the growth behavior of *Lacticaseibacillus rhamnosus GG* and the associated *pH* change in a rice-based milk alternative over a biokinetic temperature range. Integration of primary and secondary models into tertiary predictive frameworks (BR_T_ and NL_T_ models) enabled accurate forecasting of microbial growth under varying thermal conditions, with excellent statistical performance (*R*^2^ = 0.991, *RMSE* = 0.185). A parallel modeling strategy applied to *pH* dynamics further confirmed the applicability of this approach, with the tertiary *pH* model (GM_pH, T_) achieving strong predictive power (*R*^2^ = 0.941, *RMSE* = 0.386). Importantly, identified cardinal temperatures and kinetic parameters offer valuable insights for optimizing fermentation conditions in plant-based matrices. These findings underscore the potential of *L. rhamnosus* GG as a functional starter culture in the development of fermented, dairy-free probiotic beverages and provide a predictive toolset for industrial-scale process design and quality control. The correction factor could be a promising approach for evaluating the nutritional potential of various alternative plant materials used in fermentation with specific bacterial cultures. The data set, comprising nine duplicate growth curves, provides a valuable baseline for understanding LGG behavior in rice beverages. Future work should expand the dataset, include a wider range of initial cell concentrations, and evaluate additional probiotic strains across different plant-based substrates to strengthen model validation and broaden applicability.

## Data Availability

The raw data supporting the conclusions of this article will be made available by the authors, without undue reservation.

## Supplementary material

Supplementary Figure S1 Delay in the *pH* lag phase, compared to the growth lag phase of *L. rhamnosus GG* across a range of temperatures.
