# Supplementary figures and images for: Kinetic data analysis for probiotic Lacticaseibacillus rhamnosus GG growth and pH drop in rice-based milk alternative
Source: Front Microbiol. 2026 Apr 24;17:1791223. doi: 10.3389/fmicb.2026.1791223 (PMC13153081; doi:10.3389/fmicb.2026.1791223)

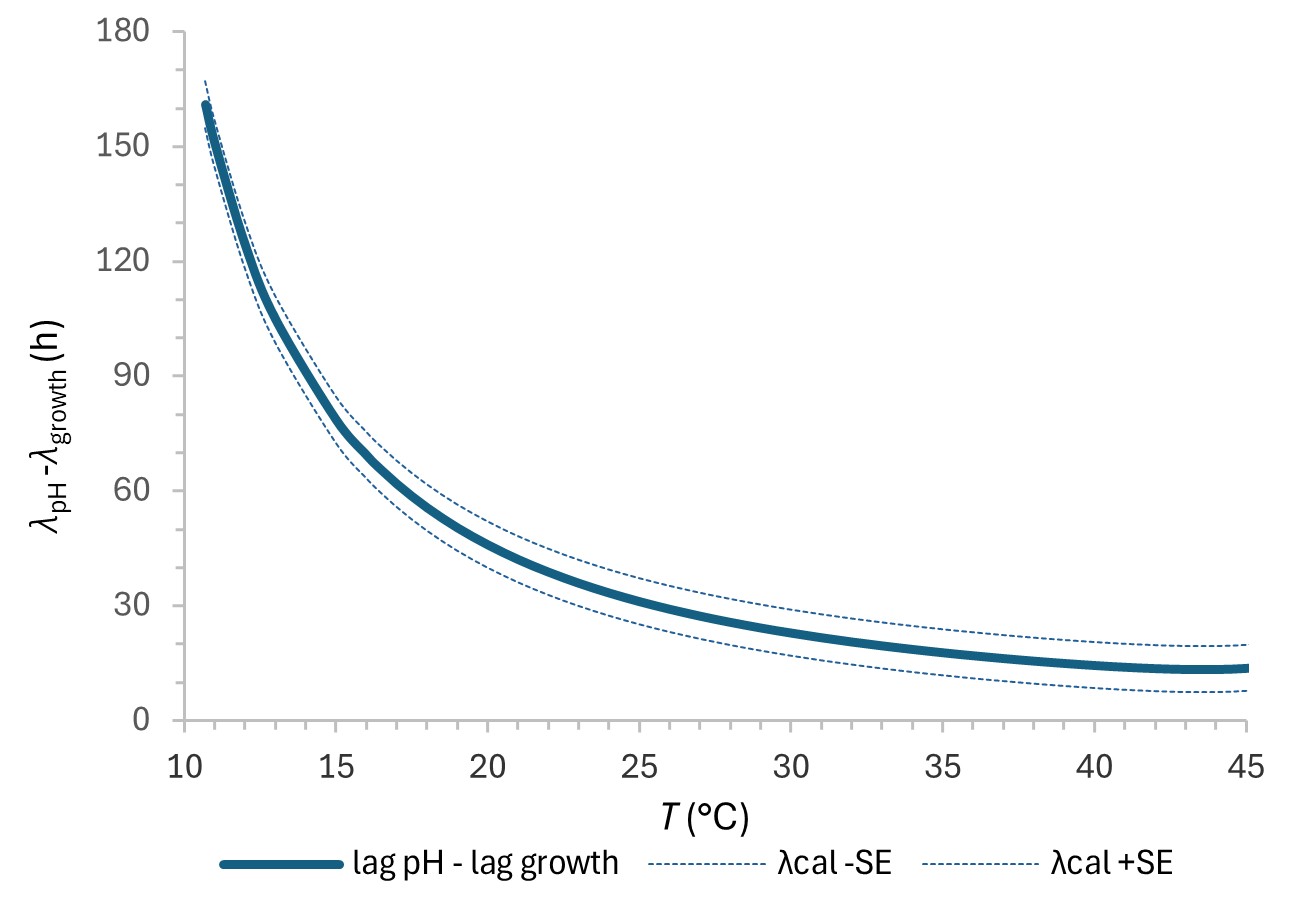

Supplement: Supplementary Figure S1 — Delay in the pH lag phase, compared to the growth lag phase of L. rhamnosus GG across a range of temperatures. [file Image_1.JPEG]
